# The impact of logo-therapy on disease acceptance and self-awareness of patients undergoing hemodialysis; a pre-test-post-test research

**DOI:** 10.1186/s12888-022-04295-2

**Published:** 2022-10-31

**Authors:** Fatemeh Zarezadeh Mehrizi, Samaneh Bagherian, Ali Bahramnejad, Zohreh Khoshnood

**Affiliations:** 1 Msc of Nursing, Department of Nursing, School of Nursing and Midwifery, Kerman University of Medical Sciences, Kerman, Iran; 2 Department of Operating Room, School of Paramedical Sciences, Birjand University of Medical Sciences, Birjand, Iran; 3 Neuroscience Research Center, Kerman University of Medical Sciences, Kerman, Iran; 4 Department of Public Health Nursing, Nursing Research Center, Razi Nursing and Midwifery Department, Kerman University of Medical Sciences, Kerman, Iran

**Keywords:** Logo-therapy, Acceptance, Self-awareness, Hemodialysis

## Abstract

**Background:**

Acceptance is considered a key variable in chronic diseases such as chronic renal failure. To achieve adaptation and acceptance, people face obstacles, and identifying these obstacles primarily requires self-awareness. logo-therapy can help a person achieve this goal. To this end, this study aimed to investigate the effect of logo-therapy on disease acceptance and self-awareness of patients undergoing hemodialysis referred to a medical centers supervised by Kerman medical science university in 2021.

**Methods:**

This quasi-experimental study was conducted with a pre-test-post-test research design on 80 patients with chronic renal failure undergoing hemodialysis in Kerman. The patients who met the inclusion criteria were selected using convenience sampling and were then randomly assigned to intervention and control groups (40 patients in each group). The logo-therapy training program was performed for the participants in the intervention group during 4 sessions of 45–60 min, individually and three times a week. The instruments used for data collection were a demographic information questionnaire, the Acceptance of Illness Scale (AIS), and the Self-Awareness Outcomes Questionnaire (SAOQ). The collected data were analyzed using SPSS software (version 22).

**Results:**

The analysis didn’t show a significant difference in the acceptance of illness scores in the intervention group before and after the intervention (P > 0.05). Besides, a statistically significant difference was observed between the intervention and control groups in terms of self-awareness, indicating that the intervention improved the dialysis patients’ self-awareness (P < 0.05). Also, the patients’ age, marriage length, and the number of children had statistically significant correlations with self-awareness and acceptance (P < 0.05).

**Conclusion:**

The results of this study showed that meaning therapy intervention can positively affect disease acceptance and self-awareness of patients undergoing hemodialysis. Since logo-therapy has been effective in other patients and the patients in this study, health officials and managers are recommended to use this intervention method to promote disease acceptance and self-awareness in these patients.

## Introduction

Chronic kidney disease is a major health problem worldwide [1]. Hemodialysis is one of the most common and important treatments for patients with chronic renal failure [2]. This is a treatment technique that replaces the activity of the kidneys [3]. According to reports published in the United States, there are more than 400,000 dialysis patients with an average age of more than 60 years, and the number of patients undergoing dialysis was over 600,000 in 2020 [4]. patients undergoing hemodialysis frequently report some psychological problems such as Depression and hopelessness, Anxiety and stress, and poor quality of life [5, 6]. Other psychological complications of the illness and its treatment are suffering from a long course of treatment. So that, they should use more coping and emotion regulation strategies [7]. Researches showed that, when people control their stress, they focus more on problem management, but when they are unable to control it, they will resort to emotion regulation [8].

Acceptance is recognized as one of the important factors in psychosocial adjustment in the final stage of kidney disease [9]. This is one of the basic elements in psychosocial adaptation to chronic diseases and disabilities [10]. Acceptance has been defined as the experience of events without any defense [11].So that, they can accept the events in the present moment [12]. The level of acceptance of the disease makes it possible to determine the severity of the patient’s emotions and negative reactions [13]. based on the researches, acceptance leads to desirable health outcomes, such as lowering the level of depression, reducing coping with maladaptation, improving adjustment coping, and improving quality of life and psychosocial adjustment, which requires the use of self-regulatory strategies [14]. The patients firstly need to identify an obstacle as an intervening force [15, 16], and identifying an obstacle primarily requires self-awareness [17].

Several studies have addressed the factors affecting the acceptance of patients. Keogh and Feehally found that the level of acceptance is shown to be higher among transplant patients than dialysis patients, and it’s related to demographic characteristics, such as age and ethnic background and transplant failure [18]. Chan et al. examined the role of acceptance together with other psychosocial factors in the adaptation process of long-term dialysis patients. These results may suggest that the effect of acceptance can be better accounted for by other psychosocial factors or that acceptance may not be important to patients who have been on dialysis for long time [19]. Chan’s et al.‘s study suggested that the effects of acceptance may not be universal across the course of disease and treatment may be more important in some stages of the disease and treatment than in other stages [20]. They supported the importance of acceptance in different stages of CKD and shows that acceptance is a mediator for other psychosocial factor [20].

Self-awareness is divided into private and public dimensions. In private self-awareness, one focuses on his feelings, thoughts, and behaviors, but in public self-awareness, one focuses more on the observable aspects of self, such as movements and appearance [21]. Overall, self-awareness is closely related to goal monitoring. goal monitoring is a way to help individuals identify barriers while pursuing a goal [22]. Self-awareness is an automated process in which a person compares their current actions with their internal standards and goals and makes changes as necessary to reduce incompatibility [23]. Self-awareness skill is one of the most important key skills a person must possess [24]. To define self-awareness, it could be said that it is the knowledge and understanding of a person about him/herself. In other words, self-awareness includes our recognition of ourselves, and the ability to be self-aware and developing self-awareness means that the person has a clear picture of his/her characteristics, values, attitudes, interests, and needs. Self-awareness is the realistic examination of beliefs, values, emotions, ideals, and potential and current abilities of ourselves, and using them in decision makings so that it benefits ourselves and our relationships with others. In fact, self-awareness is a skill that leads to a developed feeling of self-esteem. Awareness of self is, in fact, a continuous and inferential process through which we realize we exist [25].Self-awareness means gaining awareness about and recognition of the components of our existence – recognition of components such as our physical characteristics, feelings, thoughts and beliefs, values, goals, inner speech, and weaknesses and strengths [26].

The study of Devraj et al. (2008) examined Chronic Kidney Disease awareness in patients with CKD stages 1–4 in a nephrology specialty clinic and as expected, found that awareness of having CKD was high among patients in this outpatient nephrology specialty clinic [27]. The results of several studies showed that chronic kidney disease awareness was significantly higher with worse renal function. This relationship remained significant even after controlling for demographics and length of time attending clinic [27–30].

Given the complications of hemodialysis treatment, it seems that a psychological intervention for patients with chronic diseases helps them to accept their disabilities, learn to cope better with their disease, and adapt to its physical, psychological, and social consequences [31]. logo-therapy (meaning therapy) is one of these interventions [11]. Logotherapy focuses on the meaning of life and trying to find meaning is one of the most powerful motivating factors [12]. One of the main effects of logotherapy is to create hope in the person, which is positive motivation and focuses on the main purpose and meaning of life [32]. Finding meaning in life is a difficult process and happens when a person is highly motivated to do it [33]. In logo-therapy, the client is encouraged to take responsibility for their own decisions about how to live in this world, to take action, and then to decide how they can exist differently [34]. It follows an existential approach that has a philosophical and sometimes spiritual basis that can be applied in a group manner. Existential attitude is a dynamic attitude that emphasizes four basic principles derived from human existence: Death, freedom, loneliness, and meaninglessness [35].

Nurses have significant responsibilities in improving the adaptation and comfort of hemodialysis patients. Effective planning, practice, evaluation, and nursing initiatives are critical to satisfying unmet needs [36, 37]. Hemodialysis causes patients to experience a wide range of physical, psychological, economic, and social problems[38]. One of the nursing practices that is used to increase the patient’s comfort is education and counseling to the patient, because this education allows patients to cope more effectively with the problems [37]. Most of the studies in the literature on logo-therapy have focused on cancer patients and few studies have addressed the impact of meaning therapy on dialysis patients. So, the present study aimed to explore the effect of logo-therapy on acceptance and self-awareness of patients undergoing hemodialysis referring the medical center affiliated to Kerman University of Medical Sciences in 2021.

## Materials and methods

This quasi-experimental study was conducted using a pre &post-test research design in 2021. The research setting was the dialysis ward of Shafa Hospital affiliated with the Kerman university of Medical Sciences. In the present study, all patients undergoing hemodialysis who met the inclusion criteria were selected using convenience sampling and were randomly assigned to the intervention and control groups. The inclusion criteria were not having a physical illness other than kidney failure, ability to answer the questionnaires and communicate well, not having a mental illness, and the willingness to attend training sessions. The exclusion criteria were unwillingness to attend the subsequent intervention sessions, patient death, continuation of dialysis in another medical center, migration to another city, and patient leaving the study at any time at their discretion. The sample size was estimated as 80 persons (40 persons per group) using Pocock’s formula and based on a pilot sample.

Data were collected using a demographic information questionnaire, the Acceptance of Illness Scale (AIS), and the Self-Awareness Outcomes Questionnaire (SAOQ). The demographic information questionnaire assessed the patients’ demographic information (including gender, age, education, etc. ). The Acceptance of Illness Scale (AIS) was developed by Felton et all. (1984) and validated by Juczyński in Polish [15]. The scale has 8 items that assess negative health outcomes including the limitations imposed by the disease, feeling dependent on others, decreased self-esteem, and lack of self-sufficiency. The higher the level of disease acceptance, the better the adaptation and the lower the psychological distress. Each item is scored on a Likert scale ranging from 1 to 5 (1 = Strongly Agree, 2 = Agree, 3 = Undecided, 4 = Disagree, and 5 = Strongly Disagree), with number 1 indicating poor adaptation to the disease, while number 5 showing the full acceptance of the disease. A patient’s score ranges from 8 to 40, with lower scores indicating non-adaptation to and non-acceptance of the disease. Conversely, a score of nearly 40 indicates the acceptance of the disease, which hardly shows disease-related negative emotions. The content validity of the scale was assessed in terms of item simplicity, and clarity(qualitative) and relevance (quantitative) by 10 professors of the School of Nursing and Midwifery of Kerman University of Medical Sciences. The content validity index (CVI) was equal to 1. To assess the reliability of the scale, it was administered to a pilot sample of 40dialysis patients and the estimated Cronbach’s alpha was 0.78, indicating the acceptable reliability of the instrument.

The third instrument used in this study was the Self-Awareness Outcomes Questionnaire (SAOQ) developed by Sutton in 2016 [17]. A higher level of self-awareness measured by this questionnaire indicates better adaptation and less psychological distress. The questionnaire contains 38 items, each with a statement. The items are scored on a 5-point Likert scale (1 = never, 2 = rarely, 3 = sometimes, 4 = often, and 5 = always). A patient’s score ranges from 38 to 190, showing the patient’s degree of self-awareness. A low score indicates a low level of self-awareness and, conversely, a high score indicates a high level of self-awareness in the patient [39]. The content validity of the scale was assessed in terms of item simplicity, and clarity(qualitative) and relevance (quantitative) by 10 professors. The content validity index (CVI) was equal to 0.76. To assess the reliability of the questionnaire, it was administered to a pilot sample of 40 dialysis patients, and the estimated Cronbach’s alpha was 0.78, confirming the acceptable reliability of the questionnaire.

To collect the data, first, the participants were selected from among the patients who met the inclusion criteria through convenience sampling. Then, the selected patients were placed into the intervention and control groups through simple random sampling (by coin-flipping). In the next step, the questionnaires were completed by the patients in both groups before intervention. After that, the logo-therapy training program was performed for the intervention group by a skilled psychologist. During this period, the members of the control group did not receive any intervention. At the end of the logo-therapy program, the questionnaires were completed in both groups. The program was conducted individually for each patient during 4 sessions for 45–60 min and three times a week. The content of the program presented by questions and answers, and lectures methods. The materials covered in the program focused on introducing the logo-therapy approach, definition of freedom, acceptance, responsibility, finding purpose in life, discussing life and death, overcoming despair and hopelessness, and attitudes and feelings of people in life. A summary of the topic covered in the program was presented in the last session. Given the requirements for compliance with the COVID-19 prevention protocols, the program was held individually for each patient in a well-ventilated environment with the observance of social distancing regulations.

Data analysis was done by SPSS software (Version 22) using descriptive statistics (mean, frequency, and standard deviation) and analytic statistics (independent samples t-tests, analysis of covariance (ANCOVA), Mann-Whitney U test, and Wilcoxon test). All statistical procedures were performed at the significance level of 0.05 (P = 0.05).

For examining the homogeneity of the study groups in terms of demographic and quantitative contextual variables using the independent t-test. For qualitative variables T-test and chi-square test was used. T-test and ANOVA have been used to investigate clinical information in the intervention and control groups. In order to compare the acceptance and self-awareness score in and between the intervention and control groups before and after the intervention, independent t-test and paired t-test were used. A significance level of 0.05 was considered.

## Results

Most of the participants in the control group were 56 to 65 years old. However, there was no significant difference between the two groups in terms of the demographic variables(Table 1).

**Table 1:** A comparison of the participants’ demographic characteristics in the two groups

Variable	Categories	Intervention group		Control group		t-test	P-value
		Frequency	%	Frequency	%		
Age (year)	35–45	1	2.5%	8	20%	0.006	0.768*
	46–55	18	45%	15	37.5%		
	56–65	20	50%	15	37.5%		
	> 65	1	2.5%	2	5		
Marriage length	5–10	4	10%	6	15%	1.73	0.156*
	11–20	5	12.5%	8	20%		
	21–30	13	32.5%	15	37.5%		
	> 30	26	65%	6	15%		
	Not specified	14	35%	5	12.5%		
Number of children	0	5	12.5%	8	20%	4.09	0.585*
	1	3	7.5%	7	17.5%		
	2	8	20%	7	17.5%		
	3	3	7.5%	5	12.5%		
	> 4	7	17.5%	9	22.5%		
	Not specified	14	35%	4	10%		

* Independent samples t-test

There were no significant differences between the two groups in terms of gender, education, occupation, marital status, income level, and source of income(Table 2).

**Table 2:** The frequency of the demographic variables in the two groups

Variable	Categories	Intervention group		Control group		Statistic	P-value
		Frequency	%	Frequency	%		
Gender	Male	22	55%	19	47.5%	0.006	0.326*
	Female	18	45%	21	52.5%		
Education	Lower education	10	25%	15	37.5%	1.4	0.382*
	High school diploma	14	35%	11	27.5%		
	Associate degree	13	32.5%	7	17.5%		
	Bachelor’s degree	3	7.5%	7	17.5%		
Occupation	Unemployed	2	5%	4	10%	1.84	0.600*
	Worker	4	10%	5	12.5%		
	Employee	17	42.5%	8	20%		
	Self-employed	12	30%	9	22.5%		
	Retired	5	12.5%	6	15%		
	Housewife	0	0%	7	17.5%		
	Others	0	0%	1	2.5%		
Marital status	Married	30	75%	33	82.5%	1.74	0.601*
	Single	10	25%	7	17.5%		
Income level	Poor	16	40%	16	40%	1.84	0.377*
	Moderate	40	35%	22	55%		
	High	10	25%	2	5%		
Source of income	Salary	14	35%	23	57.5%	0.6	0.255*
	Others	26	65%	18	42.5%		

* Chi-square test

The results indicated that there was no statistically significant relationship between any of the clinical variables with self-awareness and acceptance (P < 0.05).

Also, the results indicated significant differences in the acceptance scores of the participants in the control group before and after the intervention and also between the control and intervention groups. However, there were no significant differences in the acceptance scores of the participants in the intervention group before and after the intervention (Table 3).

**Table 3:** A comparison of the participants’ acceptance scores in the two groups

Variable	Stage	Intervention group		Control group		P-value
		Mean	SD	Mean	SD	
Acceptance	Before the intervention	27.12	5.41	20.95	1.46	0.004
	After the intervention	14.65	5.27	19.90	1.56	0.319

The results showed significant differences in the self-awareness scores of the participants in the control group before and after the intervention and also between the control and intervention groups. Moreover, there were significant differences in the self-awareness scores of the participants in the intervention group before and after the intervention, confirming the effectiveness of the intervention program in improving the patients’ self-awareness (Table 4).

**Table 4:** A comparison of the participants’ self-awareness scores in the two groups

Variable	Stage	Intervention group		Control group		P-value
		Mean	SD	Mean	SD	
Self-awareness	Before the intervention	103.95	8.88	79.37	7.15	0.000
	After the intervention	108.12	3.16	162.72	14.92	

The results showed that the participants’ age, marriage length, and the number of children had significant correlations with self-awareness and acceptance (P < 0.05). In other words, the older participants who had been married for a longer period or had more children had higher self-awareness and acceptance. Moreover, the demographic variables had no statistically significant relationship with self-awareness and acceptance as indicated by the independent samples t-test (P < 0.05).

## Discussion

The present study evaluated the effect of logo-therapy on disease acceptance and self-awareness of patients undergoing hemodialysis. The results indicated that logo-therapy intervention can positively effect on disease acceptance and self-awareness in patients undergoing hemodialysis. logo-therapy teaches people to change their attitudes and beliefs about a situation such as the pain and suffering of an illness [40]. Asagba et al. (2015) argued that one of the main personality traits can explain the whyness and howness of behaviors in the face of unsolvable problems or challenging situations [41]. Steger et al. (2009) also showed that the meaning of life is a valuable variable in determining the mental health of individuals. The presence of meaning plays an essential role in the development and physical and mental well-being [42].

Patients with chronic disease may have difficulty with finding meaning of life. For this reason, studies have used meaning therapy intervention for these patients. Asghari et al. (2012) and Spek et al. (2008) reported that group meaning therapy intervention led to the improvement of depression in older adults [43, 44]. Mohabbat-Bahar et al. (2014) also showed that group logotherapy could reduce anxiety in women with breast cancer [45]. Delavari et al. (2014) concluded that meaning therapy can reduce anxiety and depression among mothers of children with cancer [46]. Taheri et al. (2020) showed the positive effect of group logo therapy on reducing the.

burden of hemodialysis patients’ caregivers [47]. Although the reviewed studies were different from the present study in terms of the research populations and objectives, all of them have confirmed the positive effect of logo therapy in different patients.

The data in the present study showed significant differences in the acceptance scores of the participants in the control group before and after the intervention and also between the control and intervention groups. However, there were no significant differences in the acceptance scores of the participants in the intervention group before and after the intervention. A review of the literature showed there was no study on the effect of logo therapy on acceptance in hemodialysis patients. Akbarinejhad et al. (2021) showed that treatment based on acceptance, commitment, and meaning therapy leads to the acceptance of the disease and conscious exposure to death anxiety and psychological well-being in AIDS patients[48]. Ghorbannezhad et al. (2015) also found that group meaning therapy could be an effective intervention in reducing anxiety and enhancing disease acceptance in blind and visually impaired people[49]. Haghdoost et al. (2021) highlighted that meaning therapy affects death anxiety, pain catastrophizing, pain acceptance, and pain intensity in patients with prostate cancer [50]. Mohammadi and Kamali (2018) showed that group meaning therapy can improve social adjustment in older adults [51]. Beyrami et al. (2016) also showed that group meaning therapy can help patients with leukemia to use effective coping styles and improve their adaptation to the disease[52]. Dezutter et al. (2013) showed that meaning in life may be related to the general health of chronic patients and their acceptance of their condition[53].

Other studies have been conducted in the field of accepting the disease and its impact on the satisfaction of patients with chronic diseases, health outcomes and their quality of life [20, 54–58]. Siregar and Rhamayani (2019) conducted a study with the aim of examining self-acceptance of chronic caused failure in patients who have a hemodialysis in Medan. The results of the study obtained data that (11.3%) people were at high self-acceptance, (75.5%) people were at moderate self-acceptance, and (13.2%) people were at low self-acceptance. Based on the results of the study it can be concluded that patients with chronic renal failure undergoing hemodialysis have a good self-acceptance [59]. Also, the results of this study show that usually hemodialysis patients who suffer from this disease and undergo hemodialysis treatment for a long time feel acceptance and self-awareness about the situation. Good acceptance does not mean that patients do not feel negative affect at all, but fewer patients feel negative affect and more often feel positive affect and are satisfied with the life they live. Respondents who often feel positive affect usually get support from family or closest people such as being accompanied during hemodialysis. It will be able to provide good coping for respondents [59].

Although the reviewed studies had some differences in research design, research procedure, meaning therapy sessions, and the number of sessions, they showed a good agreement in terms of their findings. The present study also confirmed the effectiveness of meaning therapy for dialysis patients. However, as the impact of meaning therapy was not significant, due to the chronic nature of the disease and the fact that the patients have to undergo hemodialysis at least three days a week and suffer many complications, they are psychologically more depressed and hopeless, so it may be less affected by therapeutic intervention than other studies. be placed According to this article, it may be necessary to organize more interventions and treatment sessions for them. So this intervention needs to be further addressed in future studies.

The data in the present study showed significant differences in self-awareness of the patients in the control and intervention groups before and after the intervention. These findings confirmed the effectiveness of meaning therapy in improving the self-awareness of dialysis patients. However, a review of the literature found no study addressing the effect of meaning therapy on self-awareness in hemodialysis patients. It was not found that the effect of meaning therapy on the self-awareness of the disease in hemodialysis patients has been investigated, so to explain this part of the results of the present study, the studies that were most consistent with the results of the present study were used.

Yahyazadeh Jeloudar et al. (2018), conducted a study titled “The Effect of Self-awareness Rising on Identification and Responsibility among High School Students”. Results have shown that self-awareness training can affect responsibility and identification among students; therefore, authorities should attempt to take necessary measures to teach self-awareness to high school students [60]. This study was deemed noteworthy since it discusses the importance of self-awareness in personal life. However, the two studies are different in their statistical population, the goal of conducting the research, the instruments used, and the way the research was conducted.

Rahmani et al. (2020), examined “Comparison of the effectiveness of self-awareness and problem-solving skills on the resilience of female employees of Kish Island”. The results showed that self-awareness skill training is effective on female employees’ resiliency. Problem-solving skills training was also effective on female employees’ resiliency. Furthermore, the results showed that self-awareness skills training increases resiliency more than problem-solving skills training. Therefore, in improving resiliency, self-awareness skill training is more effective than problem-solving skill training [61]. This research was deemed noteworthy since it discusses the importance of self-awareness in personal life. However, the two studies are different in their statistical population, the goal of conducting the research, the instruments used, and the way the research was conducted.

Shahmohamadi (2020), conducted a study with the goal of “Self-awareness skills training on self-regulation and responsibility of nursing students of University of Medical Sciences”. The results showed that self-awareness skills training has a positive effect on students’ self-regulation and responsibility. Therefore, it is concluded that self-awareness skill can be used to improve the self-regulation and responsibility of these students and they can be used as practical, accessible, and low-cost methods for increasing self-regulation and responsibility as training for nursing students [62]. This research was deemed noteworthy since it indicated that improving self-awareness can improve the self-regulation and responsibility of students. The researcher cited this paper since self-regulation and responsibility in patients undergoing hemodialysis can be effective in the process of adjusting to the illness for them. Nevertheless, the two studies are different in their statistical population, the goal of conducting the research, the instruments used, and the way the research was conducted.

Rasheed et al. (2019), conducted a study titled “Self-awareness in nursing: A scoping review”. This review delineated theoretical, educational, and personal strategies for nurses to improve their self-awareness and indicated that engagement in self-awareness at relational and contextual levels is essential for developing nurse-patient relationships [63]. This research was deemed noteworthy since it studied self-awareness in nurses. Since nurses are directly in contact with patients, improving their self-awareness can directly affect patients. However, it must be stated that the goal of conducting the research, the way the research was conducted, the instruments used, and the population of Rasheed et al. study are different from that of this study.

Carden et al. (2021) Conducted a study titled “Defining Self-Awareness in the Context of Adult Development”. The analysis of this study indicated that, in management education, self-awareness can be studied from two points of view: intrapersonal and interpersonal. The authors of this article stated that self-awareness combines these two views. Findings indicate that conscious effort is needed to develop self-awareness. [64]. This research was deemed noteworthy since it defined and studied self-awareness, and it could give the author a proper understanding of self-awareness. The goal of this article is, however, from that of this research.

Following these findings, Frankel (1984) believed that human beings are provoked by something called “desire for meaning”. He argued that life should have meaning even in the worst possible circumstances, and that life is motivated by finding its meaning. In fact, this meaning gives man courage and reduces the anxiety of life. When people are confronted with special situations and find that their lives have become meaningless, they should seek to discover new meanings for their own lives to come up with renewed motivation and enthusiasm for survival[65]. Accordingly, previous studies (Sarvarian et al., 2015; Amani et al., 2015; Amini et al., 2014; Sayyah et al., 2014) [66–69] stated that meaning therapy training has a positive and significant effect on people’s self-esteem. Furthermore, Tajlifar et al. (2021) showed that group meaning therapy intervention was effective in increasing self-confidence and reducing feelings of worthlessness in older adults with type 2 diabetes[40]. Since self-belief is part of self-awareness, these findings were in line with the data in the present study.

The findings of the present study also confirmed that the dialysis patients’ age, marriage length, and the number of children had significant correlations with self-awareness and acceptance. In other words, the older participants, those who had been married for a longer period, or those who had more children had a higher level of self-awareness and acceptance. Tran et al. (2019) showed that self-awareness has a significant relationship with the education and living conditions of students, but it had no significant correlation with gender [70]. White et al. (2018) also showed no difference between gender and self-awareness [71]. Hamedani et al. (2015), Nguyen (2019), and Huynh (2017) also highlighted the relationship between education and self-awareness in students [72–74]. These findings were not consistent with the results of the present study. These contradictory findings could be attributed to the differences in the research samples and the nature of the disease. However, to the best of the researchers’ knowledge, no study has addressed the relationship between demographic variables with self-awareness and acceptance in hemodialysis patients or other patients.

This study was conducted with some limitations. For instance, the physical and mental fatigue of the patients affected their response rate. To overcome this problem, the researcher tried to have the questionnaires completed by the participants when they were in good mental and emotional conditions.

## Conclusion

The finding of the present study indicated that meaning therapy intervention can positively affect disease acceptance and self-awareness of patients undergoing hemodialysis. In general, since hemodialysis patients face several psychological problems, interventional techniques can be used to alleviate these problems to some extent. Since training interventions such as meaning logo-therapy have an effective role in the decreasing psychological distress in hemodialysis patients. health officials and managers can use this intervention to solve the problems of hemodialysis patients and improve their self-awareness and acceptance. However, as the impact of meaning therapy was not statistically significant in this study, this intervention needs to be further addressed in future studies.

## Data Availability

All data generated or analysed during this study are included in this published article.
